# Obesity and socioeconomic disadvantage in midlife female public sector employees: a cohort study

**DOI:** 10.1186/s12889-017-4865-8

**Published:** 2017-10-24

**Authors:** Aapo Hiilamo, Tea Lallukka, Minna Mänty, Anne Kouvonen

**Affiliations:** 1 Department of Social Research, University of Helsinki, Helsinki, Finland; 2 Department of Public Health, University of Helsinki, Helsinki, Finland; 3 Finnish Institute of Occupational Health, Helsinki, Finland; 4 SWPS University of Social Sciences and Humanities in Wroclaw, Wroclaw, Poland; 5 UKCRC Centre of Excellence for Public Health (Northern Ireland), Queen’s University Belfast, Belfast, UK; 6 Administrative Data Research Centre - Northern Ireland (ADRC-NI), Queen’s University Belfast, Centre for Public Health, Institute of Clinical Sciences, Grosvenor Road, Belfast, BT12 6BJ UK; 7 Unit of Research, Development and Innovation, Laurea University of Applied Sciences, Vantaa, Finland

**Keywords:** Obesity, Social inequalities, Poverty, Midlife, Longitudinal studies, Socioeconomic disadvantage, Older workers

## Abstract

**Background:**

The two-way relationship between obesity and socioeconomic disadvantage is well established but previous studies on social and economic consequences of obesity have primarily focused on relatively young study populations. We examined whether obesity is associated with socioeconomic disadvantage through the 10–12-year follow-up, and how obesity-related socioeconomic inequalities develop during midlife among women.

**Methods:**

Baseline data were derived from the female population of the Helsinki Health Study cohort, comprising 40–60 –year-old employees of the City of Helsinki, Finland in 2000–2002 (*n* = 6913, response rate 69%). The follow-up surveys were carried out in 2007 (*n* = 5810) and 2012 (*n* = 5400). Socioeconomic disadvantage was measured by five dichotomous measures. Repeated logistic regression analyses utilising generalized estimating equations (GEE) were used to test the association between baseline self-reported obesity and the likelihood of socioeconomic disadvantage through all phases. The effect of time on the development of inequalities was examined by time interaction terms in random effect logistic regression models.

**Results:**

After adjustment for educational level, baseline obesity was associated with repeated poverty (OR = 1.23; 95% CI; 1.05–1.44), frequent economic difficulties (OR = 1.74; 95% CI; 1.52–1.99), low household net income (OR = 1.23; 95% CI; 1.07–1.41), low household wealth (OR = 1.90; 95% CI; 1.59–2.26) and low personal income (OR = 1.22; 95% CI; 1.03–1.44). The differences in poverty rate and low personal income between the participants with obesity and participants with normal weight widened during the follow-up. Living without a partner and early exit from paid employment explained the widening of inequalities.

**Conclusions:**

Weight status inequalities in socioeconomic disadvantage persisted or widened during the late adulthood.

**Electronic supplementary material:**

The online version of this article (10.1186/s12889-017-4865-8) contains supplementary material, which is available to authorized users.

## Background

Obesity is one of the leading public health problems throughout the developed world [1]. Obesity can be defined as a condition with excessive and unhealthy amount of adipose tissue in human body [2], and it is well established that obesity can impair both physical [3] and mental health [4]. The key biological mechanisms between obesity and ill health are problems with insulin and glucose tolerance, and cardiac functioning, as well as sleep-breathing abnormalities [2]. Particularly in women, obesity is also socially harmful due to its stigma, which affects the lives of women with obesity in various settings including workplace [5]. A number of studies have observed the relationship between obesity and socioeconomic disadvantage [6–17]. Moreover, previous studies have shown that women are more vulnerable to negative socioeconomic consequences of obesity than men [12–17]. The two-way relationship between obesity and socioeconomic status is illustrated by the fact that cumulative social and economic disadvantages expose individuals to weight gain and obesity [18, 19].

In women obesity is associated with various educational and labour market outcomes, such as lower education [20], higher rates of unemployment [21] and work disability [22, 23]. Intervention studies suggest that the link from obesity to work disability is strong and robust, although work disability may also expose to weight gain through unhealthy behaviours such as physical inactivity and poor diet [23]. Obesity has been shown to have a negative effect on personal [24, 25] and household income in women [26, 27]. Furthermore, several longitudinal studies have shown a link between early life obesity and adulthood socioeconomic disadvantage [12–17]. In a British longitudinal cohort study, women with persisting obesity from childhood to the age of 30 were less likely to be gainfully employed and having a current partner [12]. A study of US adults found that women who were persistently overweight through early adulthood were more likely to receive social benefits and not to have further education qualifications or current partner at the age of 40 [13]. In addition, in cross-sectional studies obesity has been associated with economic difficulties independently of traditional socio-economic measures [27, 28]. Whilst self-reported economic difficulties are closely correlated with most commonly used indicators of socioeconomic disadvantage, these concepts are not interchangeable [27].

Although previous work has expanded our understanding of the two-way relationship between obesity and socioeconomic disadvantage, a number of gaps still remain. Prior research has mainly focused on relative young populations and therefore has not fully captured the effect of work disability on socioeconomic inequalities. Moreover, the earlier studies have usually measured socioeconomic disadvantage only at one time point and have not taken into consideration how disadvantages develop in midlife and later [12–16]. There are also fewer studies focusing on the association between obesity and socioeconomic disadvantage in midlife women. It is important to focus on later parts of life course because even if the obesity levels have risen in all age groups, the prevalence of obesity is particularly high in midlife and older adults [29]. Since the association between obesity and low socioeconomic status is more robust in women, a closer look particularly at women in later life is justified [7].

**Fig. 1:**
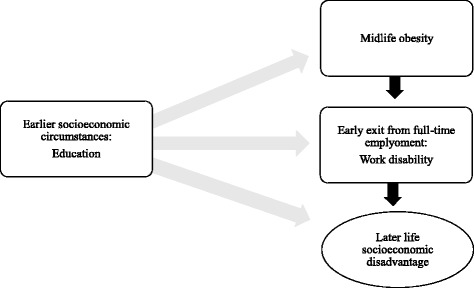
The association between midlife obesity and socioeconomic disadvantage

This study aimed to fulfil these gaps by examining the relationship between baseline obesity and five indicators of socioeconomic disadvantage in midlife women; and investigating how these inequalities change over a 10-to-12-year follow-up. Based on the earlier literature on the association between obesity and socioeconomic status, we hypothesised that obesity in women is linked to socioeconomic disadvantage in later life; and that the course of inequalities change over time. In midlife women with obesity may face higher risks of work disability [22, 23] and early exit from full-time employment, which can strengthen the association between obesity and socioeconomic disadvantage (Fig. 1).

## Methods

### Data

The data were derived from the Helsinki Health Study, which examines the health and well-being of the staff of the City of Helsinki (capital of Finland) in later adulthood. The eligibility criteria for being part of the study was to have a job contract with the City of Helsinki in 2000, 2001 or 2002 and turning 40, 45, 50, 55 or 60 in one of those years. More than 90% had permanent job contracts, and the annual turnover is ca 10%. Employees were working in general local administration, public transport, technical services, education and culture as well as health and social care sector. In the current study, we included only female participants due to the gendered nature of obesity [5, 7, 16], and the fact the original study population was female-dominated due to the occupation structure in the Finnish municipal sector [30]. Baseline (Phase 1) data were collected by postal surveys in 2000, 2001, and 2002 and the target sample included 10,343 female employees. About 69% (*n* = 7154) of these employees returned the baseline questionnaire. The first follow-up survey was collected in 2007 (Phase 2), when the response rate was 84% (*n* = 5857). In the second follow-up in 2012 (Phase 3) the response rate was 79% (*n* = 5450). Previous non-response analysis from the present cohort has shown that respondents represent the whole study population adequately [30].

We excluded those with missing value for height and/or weight (*n* = 88), those who were pregnant (*N* = 33), and those who were underweight (body mass index, BMI <18.5, *n* = 90) at baseline. After these exclusions, the study population consisted of 6913 participants at Phase 1, 5810 at Phase 2, and 5400 at Phase 3. Also, in each phase there were some data loss due to missing values in our disadvantage measures. At baseline, 78% of the participants provided an informed consent for linking employers’ personnel register data to their survey responses. For this subgroup (baseline *n* = 4629), we extracted register data on personal income and annual working hours for the time these participants remained employed at the City of Helsinki.

### Socioeconomic disadvantage

We conceptualised socioeconomic disadvantage as a multidimensional combination of low relative and subjective material socioeconomic positions. The rationale for focusing on material aspects was the fact that these dimensions of socioeconomic position are more recent than educational aspects for midlife and older adults. To keep our analyses comparable with previous studies, suitable for the study population of older adults and still multidimensional we measured socioeconomic disadvantages by five dichotomised indicators: low household income, household income below poverty line, low personal income (in the subgroup of those who consented to record linkage), self-reported economic difficulties, and low household wealth. These variables were measured at each Phase. Net household income was measured with a multicategorical (seven categories in 2000–2007 and nine categories in 2012) question ranging an average from less than 1300€ to more than 5000€ a month and was re-coded using the middle point of each income category. Low household income was defined as being in the lowest household income quartile among the study population. We used the lowest quartile as a cut-off point to focus on relative material disadvantage within households of the study population and to keep our results comparable with studies using income quartiles as measures of socioeconomic status. Income below poverty line was also measured by self-reported household income. We used the standard measure of poverty, 60% below national equalised income [31]. Low personal income was defined as having an annual salary in the lowest fifth of the salaries of female participants of the cohort in each Phase using reliable register based measures that eliminated potential reporting bias. This measure examines disadvantage indicated by relative low personal earnings.

The indicator of self-reported economic difficulties was constructed from five-categorical questions “*How often do you have enough money to buy the food or clothing you or your family need?*” ranging from *“never”* to *“always*” and “*How much difficulty do you have in meeting the payment of bills?*” ranging from *“very little”* to *“very much”.* Those who reported having “*always or usually money to buy food and clothing*” and “*never or rare have difficulties in paying bills*” was categorised as having “*no economic difficulties*”. Those who reported to have “*less often money for food and clothing”* or more often had *“difficulties in paying bills*” were defined to have “*frequent economic difficulties”* [32, 33]. This measure was constructed to investigate subjective dimensions of socioeconomic disadvantage providing a valuable addition to material resource based measures. Furthermore, we analysed the relationship between baseline obesity status and low household wealth although wealth data were available only from Phases 2 and 3. Household wealth was measured in survey by self-reported total value of household possessions including real estate and vehicles, with debts and loans (ten categories) subtracted and then weighted by possible partner in same household. Dichotomous low net household wealth variable was formed using the lowest quintile as a cut-off point to get an exact measure of relative low cumulative income among study population. Our wealth indicator measures long-term accumulated wealth and is thus a more stable measure than personal income measures, which are prone to random shocks and potential fluctuation.

### Weight status

BMI was calculated from self-reported weight and height from baseline [weight (kg) divided by height (m)^2^] in a standard way. Baseline weight status categories were normal weight (BMI 18.50–24.99), overweight (BMI 25.00–29.99) and obese (BMI >30.00) [34].

### Covariates

Marital status, full-time employment, educational level and age were used as a covariates; the first two as time variant. Marital status was defined by being married or cohabiting vs others. Full-time work was defined by asking whether participants were in full-time employment or not. Those who were not in full-time employment were either on disability, part-time or old-age pension, on long-term sick leave, in part-time work or unemployed. Noteworthy, unemployment and part-time work were marginal in our sample (less than 1% and 2%, respectively, in the follow-ups) [30]. Baseline educational level had three categories: compulsory education only; secondary education; and higher education. e.g. university degree.

### Statistical analysis

The analysis was conducted in three stages. As a first, descriptive step, we ran cross-tabulations analysing types of socioeconomic disadvantage by weight status. Weight status categories were compared in each outcome and Phase after adjusting for age. We then used repeated measures of socioeconomic disadvantage in generalized estimating equations (GEE) logistic regression analyses. GEE estimates parameters over the population and employs working correlation matrix that takes into account the unknown correlation between within-subject measures [35]. We used exchangeable correlation structure since alike correlativity was assumed between the individual’s responses [36]. Two models were built, adjusting first for age, and then additionally for time variant marital status. Corrected Quasi Likelihood under Independence Model Criterion (QICC) values were used to demonstrate differences in fit of each model of each disadvantage indicator after adding further adjustments.

The third step was to examine the changes in the indicators of disadvantage through the follow-up period utilising random effect (random intercept for subjects) logistic regression with time by baseline weight category interaction (baseline weight category*Phase). In contrast to population- average GEE models used in the second step, random effect logistic regression models investigated individual level changes in socioeconomic disadvantage measures [37]. Group by time interaction models demonstrate whether or not the follow-up time changes in disadvantage measures are similar between the baseline weight categories. We used Phases as a measure of time to investigate if the socioeconomic disadvantages between weight status groups had changed during the follow-up period. As intermediary variables marital status and employment status were tested in separate models. We reported odds ratios (ORs) and 95% confidence intervals (CI) with model *n* in second and third step of the statistical analyses. In addition, we conducted sensitivity analyses including time-variant weight status in the second step.

Statistical Package for the Social Sciences software, version 22 (SPSS Inc., Chicago,Il) and Stata (STATA Corp, College Station, TX) were used in statistical analyses.

## Results

At baseline (Phase 1), the proportions of obesity, overweight and normal weight were 14%, 32% t and 54%, respectively (Table 1). The mean age was 49 (SD, 6.54) years (48 in those with normal weight and 50 in those who were overweight or obese). The majority of participants lived with a partner (at baseline 68% of those with normal weight, 69% of those who were overweight and 64% of those who were obese). Having household income in the lowest quartile varied by weight category in each Phase and there were also inequalities by weight in self-reported economic difficulties in all Phases. Moreover, there were differences between the weight groups in having low personal income in all Phases. All in all, the participants with obesity were the most disadvantaged throughout the follow-up in terms of the examined disadvantage indicators apart from low personal income with which they differed from the participants with overweight only at Phase 3.

**Table 1:** Characteristics of the study population by baseline (Phase 1) body weight

Baseline weight	Normal weight (BMI < 25) %	Overweight (25 < =BMI < 30) %	Obese (BMI= > 30) %	Total n
Participated				
Phase 1	54	32	14	6913
Phase 2	54	32	14	5810
Phase 3	54	32	14	5400
Age (baseline)**				
40	24	15	16	1400
45	24	20	19	1500
50	22	23	20	1523
55	21	27	31	1691
60	9	15	14	797
Mean age	48.2	50.1	50.2	
Education (baseline)**				
Compulsory education only	17	26	27	1455
Secondary education	53	55	56	3724
Higher education	29	19	17	1668
*P* value				
Working full-time				
Phase 1	93	94	93	0.250
Phase 2	74	64	62	<0.001
Phase 3	56	42	37	<0.001
Married or cohabiting				
Phase 1	68	69	64	0.037
Phase 2	66	66	61	0.007
Phase 3	65	63	57	0.001
Low net household income				
Phase 1	24	26	32	<0.001
Phase 2	22	27	29	<0.001
Phase 3	20	26	29	<0.001
Below poverty line				
Phase 1	11	13	14	0,018
Phase 2	13	15	19	<0.001
Phase 3	8	11	14	<0.001
Frequent economic difficulties				
Phase 1	20	24	29	<0.001
Phase 2	18	21	29	<0.001
Phase 3	16	20	27	<0.001
Low wealth^+^				
Phase 2	19	22	29	<0.001
Phase 3	17	21	27	<0.001
Low personal income*				
Phase 1	18	22	22	0,003
Phase 2	17	24	24	<0.001
Phase 3	17	23	26	<0.001

*P* values: differences between baseline weight groups (from χ2 test)

^*^subgroup = only those who consented to record linkage and were employed by the Helsinki City throughout the study period (Phase 1 N = 4629, Phase 2 *N* = 3759, Phase 3 *N* = 2507)

^**^Total 100% in columns

+ = no data from Phase 1

As shown in Table 2 **,** in GEE logistic regression models, baseline obesity was associated with repeated measures of socioeconomic disadvantage after adjustment for age and educational level. In model 1, baseline obesity was linked to higher odds of low household income (OR = 1.23; 95% CI: 1.07–1.41), poverty (OR = 1.23; 95% CI: 1.05–1.44), frequent economic difficulties (OR = 1.74; 95% CI: 1.52–1.99), low household wealth (OR = 1.90; 95% CI: 1.59–2.26), and low personal income (OR = 1.22; 95% CI: 1.03–1.44). Being overweight at baseline was also significantly associated with a higher likelihood of frequent economic difficulties (OR = 1.21; 95% CI: 1.08–1.35), low household wealth (OR = 1.24; 95% CI: 1.08–1.43) and low personal income (OR = 1.17; 95% CI: 1.03–1.33). In Model 2, we additionally adjusted for marital status as a time variant covariate; this adjustment did not attenuate the associations.

**Table 2:** Odds ratios (95% confidence intervals) for associations between baseline (Phase 1) body weight status and socioeconomic disadvantage at Phases 1–3 among women, repeated measures analysis (GEE), the Helsinki Health Study, Finland, 2000–2012

	Model 1^a^		Model 2^b^	
Low household net income	Odds ratio	95% CI	Odds ratio	95% CI
Normal weight	1.00	ref.	1.00	ref.
Overweight	1.01	0.91–1.12	1.10	0.97–1.24
Obese	1.23	1.07–1.41	1.21	1.04–1.41
Number of subjects	*6809*		*6807*	
QICC*	*18,349.35*		*11,836.38*	
Income below poverty				
Normal weight	1.00	ref.	1.00	ref.
Overweight	1.03	0.90–1.17	1.06	0.94–1.21
Obese	1.23	1.05–1.44	1.20	1.02–1.4
Number of subjects	*6809*		*6807*	
QICC	*12,114.43*		*10,799.45*	
Frequent economic difficulties				
Normal weight	1.00	ref.	1.00	ref.
Overweight	1.21	1.08–1.35	1.23	1.10–1.37
Obese	1.74	1.52–1.99	1.71	1.50–1.96
Number of subjects	*6836.00*		*6832.00*	
QICC	*17,112.79*		*16,468.48*	
Low household wealth				
Normal weight	1.00	ref.	1.00	ref.
Overweight	1.24	1.08–1.43	1.25	1.09–1.44
Obese	1.90	1.59–2.26	1.90	1.59–2.26
Number of subjects	*5747*		*5743*	
QICC	*9268.02*		*9157.90*	
Low personal income				
Normal weight	1.00	ref.		
Overweight	1.17	1.03–1.33		
Obese	1.22	1.03–1.44		
Number of subjects	*4623*			
QICC	*10,130.52*			

*CI* Confidence interval

Weight category defined by body mass index (normal weight BMI 18.50–24.99, overweight BMI 25.00–29.99 and obese BMI >30.00)

^*^Corrected Quasi Likelihood under Independence Model Criterion (in smaller-is-better form)

^a^Adjusted for age and educational level

^b^Additionally adjusted for marital status as a time variant covariate

As presented in Table 3, compared to participants with normal weight, baseline obesity and overweight were associated with changes in some of the examined disadvantage measures. After adjustment for age and educational level, as time went by, participants with obesity were more likely to have an income below the poverty line (OR = 1.30; 95% CI: 1.07–1.59) and to have low personal income (OR = 1.40; 95% CI: 1.11–1.76) compared to participants with normal weight. Differences in low household wealth and income and economic difficulties did not change over time. In Model 2 we additionally adjusted for marital status as a time variant covariate, and the higher likelihood of negative development in poverty between those with obesity compared to those with recommended healthy weight remained statistically significant (OR = 1.25; 95% CI: 1.03–1.52). After further adjustment for employment status as a time variant covariate in Model 3, obesity became statistically non-significant. In Model 1, overweight vs. normal weight comparison showed that baseline overweight was associated with a significantly higher likelihood of negative development in low personal income (OR = 1.20; 95% CI: 1.01–1.43) and low household income (OR = 1.24; 95% CI: 1.07–1.42) although the latter association attenuated after further adjustment for marital status (Model 2).

**Table 3:** Odds ratios (95% confidence intervals) for associations of baseline (Phase 1) body weight status and the changes in socioeconomic disadvantages through Phases 1–3 among women, time by group interaction, repeated measures analysis (logistic regression with random intercept for subjects), the Helsinki Health Study, Finland, 2000–2012

	Model 1^a^		Model 2^b^		Model 3^c^	
Low household income (*time)	Odds ratio	95% CI	Odds ratio	95% CI	Odds ratio	95% CI
Normal weight	1.00	ref.	1.00	ref.	1.00	ref.
Overweight	1.24	1.07–1.42	1.15	0.99–1.34	1.04	0.89–1.22
Obese	1.14	0.95–1.37	1.02	0.84–1.24	0.91	0.75–1.12
Number of subjects	*6809*		*6807*		*6805*	
Income below poverty (*time)						
Normal weight	1.00	ref.	1.00	ref.	1.00	ref.
Overweight	1.10	0.95–1.29	1.08	0.92–1.26	0.99	0.84–1.16
Obese	1.30	1.07–1.59	1.25	1.03–1.52	1.13	0.93–1.39
Number of subjects	*6809*		*6807*		*6805*	
Frequent economic difficulties (*time)						
Normal weight	1.00	ref.	1.00	ref.	1.00	ref.
Overweight	1.03	0.90–1.18	1.01	0.88–1.16	0.98	0.86–1.12
Obese	1.17	0.98–1.39	1.15	0.97–1.37	1.11	0.93–1.32
Number of subjects	*6836*		*6832*		*6831*	
Wealth (*time)						
Normal weight	1.00	ref.	1.00	ref.	1.00	ref.
Overweight	1.11	0.75–1.64	1.09	0.74–1.61	1.11	0.75–1.63
Obese	1.40	0.85–2.29	1.35	0.83–2.21	1.36	0.83–2.23
Number of subjects	*5747*		*5743*		*5734*	
Low personal income (*time)						
Normal weight	1.00	ref.				
Overweight	1.20	1.01–1.43				
Obese	1.40	1.11–1.76				
Number of subjects	*4623*					

*CI* confidence interval

Weight category defined by body mass index (normal weight BMI 18.50–24.99, overweight BMI 25.00–29.99 and obese BMI >30.00)

^a^Adjusted for age and educational level

^b^Additionally adjusted for marital status as a time variant covariant

^c^Additionally adjusted for employment status as a time variant covariant

*interaction term

## Discussion

This study sought to examine the relationship between baseline obesity status and five key indicators of socioeconomic disadvantage in female public sector employees during midlife and later adulthood. Moreover, we examined how obesity-based inequalities developed over the follow-up. The main findings were that baseline obesity and overweight were associated with multiple indicators of socioeconomic disadvantage, and that marital status did not explain these differences. In addition, baseline obesity was associated with negative development in personal income and poverty status, and overweight was associated with negative development in low household income. However, living without a partner and early exit from employment explained adverse development in these disadvantage measures.

Earlier cross-sectional [6, 7, 10] and longitudinal [11–17] studies have shown that obesity is associated with multiple indicators of socioeconomic disadvantage such as poverty, low income and low wealth [38]. In line with this research the current study found significant weight status inequalities by low wealth, poverty status, low household income, low personal income and economic difficulties. Our results from repeated economic difficulties throughout the 10- to 12-year study period are also consistent with earlier cross-sectional results suggesting a relationship between multiple socioeconomic indicators and obesity [27, 28].

Moreover, our findings of the changes in disadvantages by baseline weight groups are in keeping with previous research. In longitudinal settings socio-economic disadvantages have been shown to persist or widen over time from childhood and adolescence [12–15]. Previous research has usually examined the effect of childhood, adolescent or persistently high BMI on the adulthood socioeconomic disadvantage, whereas this study found that also midlife BMI is associated with multiple indicators of socio-economic disadvantage in midlife and late adulthood. Previous findings have indeed indicated that weight status inequalities widen in later part of working life when the risk of disability retirement is at its highest [23].

Prior studies have presented various explanations for the two-way relationship between obesity and socioeconomic disadvantage. Disadvantage may expose individuals to weight gain and obesity due to unhealthy lifestyles [19]. However, it has also been found that obesity has negative socioeconomic consequences that are caused by weight stigma [5], lower productivity [22, 23] and health hazards [3, 4]. A further possible explanation may be that there is a third factor that contributes both to obesity and later life socioeconomic disadvantage, such as low childhood socioeconomic status [27, 39].

According to our results the effect of early exit from paid employment (mainly disability retirement or long-term sick leave) explains the differences in the development of poverty inequalities between those with obesity and those with a recommended healthy weight. Therefore, changes in disadvantage may be affected by early retirement or low pensions. However, as we found that women with obesity were more likely to drop into the lowest personal income quintile, the results indicate that weight based inequalities widened also for those who continued working for the Helsinki City. This may be due to higher rates of sick leave [22], weight based wage or promotion discrimination [5] or some other reason.

There are two main reasons why we did not include men in our analyses. Previous multidisciplinary research has demonstrated the gendered nature of obesity [7, 16]. Although in many ways the physical health effects of obesity may be quite similar in both genders, the social and psychological outcomes of obesity are likely to differ. Obesity is suggested to be more socially harmful for women in terms of social mobility [39], weight-based discrimination [5] and mental health [14]. Furthermore, due to the female-dominated public sector, our cohort had a much smaller proportion of men.

We adjusted our models for educational level. Arguably, low education exposes to various types of socioeconomic disadvantage. Consequently, adjusting models for education can potentially lead to an over-adjustment. However, we ran additional analysis without adjusting for educational level and this did not change our main results (Additional file 1: Table S1 and Additional file 2: Table S2).

### Methodological considerations

The main strength of the present study is its cohort design with three time points and relatively high response rates. In addition to surveys, we used data from employers’ personnel register which provides valid and accurate information on personal income. An additional strength is our repeated measures analysis design in which we used three different phases; this strengthens the validity of our disadvantage measures. Five different indicators were used to measure socioeconomic disadvantage. Finally, due to 10- to 12-year study period, we were able to measure change in these measures of disadvantage.

There are some limitations in the current study. First, we used self-reported weight and height (BMI) as a measure of baseline obesity; BMI is not the only measure of obesity and it is prone to inaccuracy [40], however, it is shown to be a consistent predictor of various obesity-related health outcomes [41, 42]. Second, we used BMI only from one-time point (baseline). We did not include subsequent weight status in our main analyses as our research aim was to investigate how baseline weight status is associated with the selected indicators of socioeconomic disadvantage; the use of time-variant weight category would not allow to investigate how baseline obesity can determine changes in the disadvantage measures. Nevertheless, to test the effect of possible change of weight category within the study period, we ran an additional analysis using time variant weight and repeated measures of socioeconomic disadvantages. This did not change our main results from repeated measures logistic regression analysis with GEE although using a time-variant weight status attenuated the association between obesity and the poverty measure (Additional file 3: Table S3).

Third, there are possible sources of inaccuracy in our measures of socio-economic disadvantage, the main limitation being that apart from the measure of personal income these measures came from self-reports. Since BMI was also self-reported, this can lead to common method bias. Moreover, as a measure of poverty we used national at-risk-of-poverty level at each Phase. At-risk-of-poverty level is defined by 60% of the national median income each year; [31] it is therefore a relative measure and reflects national income inequality trends. Also, there may be some uncertainly in low household income and wealth variables: distribution of these measures evolved somewhat in each Phase because of the approximate nature of multi-categorical questions. In the wealth variable, an additional source of inaccuracy is possible due to the difficulties in estimating subjectively the total household wealth from multiple pieces of information.

Fourth, our study population was comprised of midlife women at baseline who were working for a relatively secure public sector employer in Helsinki, the capital of Finland. This limits generalizability of our findings in terms of the age group, labour market status, industry and geography. Arguably, the association between obesity and socioeconomic disadvantage might have been stronger if the study population consisted additionally of employees in more precarious and less secure employment, unemployed people, and those who are outside of the labour market.

Fifth, our study period, which was 10 to 12 years, might have been too short to capture significant changes in some of the measures we used. We used dichotomous low wealth indicator as one measure of disadvantage, although the data from wealth were available only from Phases 2 and 3. Moreover, we were unable to capture the potential effect of the duration of non-marriage or non-employment status. Arguably, the effect of non-employment status may have a time lag since earnings from, for instance, savings may alleviate the effect of sudden drop of personal income. Finally, it should be noted that our results from the time interaction models are likely to be conservative. This relates to the fact that the response rates at Phases 2 and 3 were somewhat lower for those were obese and for those who reported disadvantages at Phase 1 (Additional file 4: Table S4).

## Conclusions

This study showed that weight status inequalities in socioeconomic disadvantage persist or widen in midlife and late adulthood. Policies supporting work ability of people with obesity are important in terms of tackling the widening of obesity based socioeconomic inequalities in later life.

## Additional files

Additional file 1: Table S1. Odds ratios (95% confidence intervals) for associations between baseline (Phase 1) body weight status and socioeconomic disadvantage as dependent variables at Phases 1–3 repeated measures analysis (GEE), the Helsinki Health Study, Finland, 2000–2012. (DOCX 83 kb)

Additional file 2: Table S2. Odds ratios (95% confidence intervals) for associations of baseline (Phase 1) body weight status and the changes in socioeconomic disadvantages through Phases 1–3 among women, time by group interaction, repeated measures analysis (logistic regression with random intercept for subjects), the Helsinki Health Study, Finland, 2000–2012. (DOCX 97 kb)

Additional file 3: Table S3. Odds ratios (95% confidence intervals) for associations time variant body weight status and socioeconomic disadvantage at Phases 1–3 among women, repeated measures analysis (GEE), the Helsinki Health Study, Finland, 2000–2012. (DOCX 77 kb)

Additional file 4: Table S4. Response rates at Phases 2 and 3 by baseline body weight status and indicators of socioeconomic disadvantage among women, the Helsinki Health Study, Finland, 2000–2012. (DOCX 65 kb)

## Abbreviations

BMI: Body mass index
CI: Confidence interval
OR: Odds ratio
