# Supplementary material for: Obesity and socioeconomic disadvantage in midlife female public sector employees: a cohort study
Source: BMC Public Health. 2017 Oct 24;17:842. doi: 10.1186/s12889-017-4865-8 (PMC5655943; doi:10.1186/s12889-017-4865-8)
Supplement: Supplementary file 1 — Odds ratios (95% confidence intervals) for associations between baseline (Phase 1) body weight status and socioeconomic disadvantage as dependent variables at Phases 1–3 repeated measures analysis (GEE), the Helsinki Health Study, Finland, 2000–2012. (DOCX 83 kb) [file 12889_2017_4865_MOESM1_ESM.docx]

| **Supplement table 1.** **Odds ratios (95% confidence intervals) for associations between baseline (Phase 1) body weight status and socioeconomic disadvantage as dependent variables at Phases 1-3 repeated measures analysis (GEE), the Helsinki Health Study, Finland, 2000-2012** | | | | | | | | | |
| --- | --- | --- | --- | --- | --- | --- | --- | --- | --- |
|  | **Model 1^a^** | | | | **Model 2^b^** | | | | |
| **Low household net income** | Odds ratio | 95% CI | | | | Odds ratio | | 95% CI | |
| Normal weight | 1.00 | ref. | | | | 1.00 | | ref. | |
| Overweight | 1.14 | 1.02 – 1.26 | | | | 1.27 | | 1.13 – 1.43 | |
| Obese | 1.39 | 1.22 – 1.60 | | | | 1.40 | | 1.19 – 1.63 | |
| *Number of subjects* | *6873* |  | | | | *6870* | |  | |
| *QICC** | *19297.50* |  | | | | *12887.79* | |  | |
| **Income below poverty** | | |  | | | | |  | |
| Normal weight | 1.00 | ref. | | | | 1.00 | | ref. | |
| Overweight | 1.18 | 1.04 – 1.34 | | | | 1.20 | | 1.06 – 1.37 | |
| Obese | 1.42 | 1.21 – 1.66 | | | | 1.35 | | 1.14 – 1.58 | |
| *Number of subjects* | *6873* |  | | | | *6870* | |  | |
| *QICC* | *12851.23* |  | | | | *11471.99* | |  | |
| **Frequent economic difficulties** | | | | | | |  | | |
| Normal weight | 1.00 | ref. | | | | 1.00 | | ref. | |
| Overweight | 1.32 | 1.19 – 1.47 | | | | 1.34 | | 1.20 – 1.49 | |
| Obese | 1.92 | 1.68 – 2.20 | | | | 1.88 | | 1.65 – 2.16 | |
| *Number of subjects* | *6901* |  | | | | *6896* | |  | |
| *QICC* | *17657.54* |  | | | | *17039.01* | |  | |
| **Low household wealth** | | |  | | | | |  | |
| Normal weight | 1.00 | ref. | | | | 1.00 | | ref. | |
| Overweight | 1.42 | 1.24 – 1.63 | | | | 1.43 | | 1.25 – 1.63 | |
| Obese | 2.16 | 1.83 – 2.55 | | | | 2.15 | | 1.81 – 2.54 | |
| *Number of subjects* | *5797* |  | | | | *5793* | |  | |
| *QICC* | *9836.71* |  | | | | *9715.53* | |  | |
| **Low personal income** | | |  | | | | |  | |
| Normal weight | 1.00 | ref. | | | |  | |  | |
| Overweight | 1.33 | 1.17 – 1.51 | | | |  | |  | |
| Obese | 1.39 | 1.18 – 1.62 | | | |  | |  | |
| *Number of subjects* | *4658* |  | | | |  | |  | |
| *QICC* | *10640.71* |  | | | |  | |  | |
| Socioeconomic disadvantage measures as outcome variables  CI. Confidence interval.  Weight category defined by body mass index (normal weight BMI 18.50-24.99, overweight BMI 25.00-29.99 and obese BMI >30.00) | | | | | | | | |  |
| *Corrected Quasi Likelihood under Independence Model Criterion (in smaller-is-better form). Not comparative cross disadvantage measures. | | | | | | | | | |
| a Adjusted for age. | | | |  |  | | | |  |
| b Additionally adjusted for marital status as time variant | | | | | | | | | |
