# Supplementary material for: Obesity and socioeconomic disadvantage in midlife female public sector employees: a cohort study
Source: BMC Public Health. 2017 Oct 24;17:842. doi: 10.1186/s12889-017-4865-8 (PMC5655943; doi:10.1186/s12889-017-4865-8)
Supplement: Supplementary file 2 — Odds ratios (95% confidence intervals) for associations of baseline (Phase 1) body weight status and the changes in socioeconomic disadvantages through Phases 1–3 among women, time by group interaction, repeated measures analysis (logistic regression with random intercept for subjects), the Helsinki Health Study, Finland, 2000–2012. (DOCX 97 kb) [file 12889_2017_4865_MOESM2_ESM.docx]

| **Supplement table 2**. **Odds ratios (95% confidence intervals) for associations of baseline (Phase 1) body weight status and the changes in socioeconomic disadvantages through Phases 1-3 among women, time by group interaction, repeated measures analysis (logistic regression with random intercept for subjects), the Helsinki Health Study, Finland, 2000-2012** | | | | | | | | | | | | | | | | | | | |
| --- | --- | --- | --- | --- | --- | --- | --- | --- | --- | --- | --- | --- | --- | --- | --- | --- | --- | --- | --- |
|  | **Model 1^a^** | | | | | **Model 2^b^** | | | | | | | | | | **Model 3^c^** | | | |
| **Low household income (*time)** | Odds ratio | | | 95% CI | | | | Odds ratio | | 95% CI | | | | Odds ratio | | | | | 95% CI |
| Normal weight | 1.00 | | | ref. | | | | 1.00 | | ref. | | | | 1.00 | | | | | ref. |
| Overweight | 1.24** | | | 1.08 - 1.43 | | | | 1.16 | | 0.99 - 1.35 | | | | 1.05 | | | | | 0.90 - 1.23 |
| Obese | 1.14 | | | 0.95 - 1.37 | | | | 1.01 | | 0.83 - 1.23 | | | | 0.91 | | | | | 0.74 - 1.11 |
| *Number of subjects N* | *6873* | | |  | | | | *6870* | |  | | | | *6868* | | | | |  |
| **Income below poverty (*time)** | | | | | | | | | | | |  | | | | | |  | |
| Normal weight | 1.00 | | | ref. | | | | 1.00 | | ref. | | | | 1.00 | | | | | ref. |
| Overweight | 1.11 | | | 0.95 - 1.29 | | | | 1.08 | | 0.93 - 1.27 | | | | 0.99 | | | | | 0.85 - 1.17 |
| Obese | 1.30** | | | 1.07 - 1.58 | | | | 1.24* | | 1.02 - 1.50 | | | | 1.12 | | | | | 0.92 - 1.38 |
| *Number of subjects N* | *6873* | | |  | | | | *6870* | |  | | | | *6868* | | | | |  |
| **Frequent economic difficulties (*time)** | | | | | | | | |  | | | | | | | |  | | |
| Normal weight | 1.00 | | | ref. | | | | 1.00 | | ref. | | | | 1.00 | | | | | ref. |
| Overweight | 1.05 | | | 0.91 - 1.20 | | | | 1.02 | | 0.89 - 1.17 | | | | 0.99 | | | | | 0.86 - 1.14 |
| Obese | 1.18 | | | 0.99 - 1.40 | | | | 1.16 | | 0.97 - 1.37 | | | | 1.12 | | | | | 0.94 - 1.33 |
| *Number of subjects N* | *6901* | | |  | | | | *6896* | |  | | | | *6895* | | | | |  |
| **Wealth (*time)** | |  | | |  | | | |  | | | |  | | | | | |  |
| Normal weight | 1.00 | | | ref. | | | | 1.00 | | ref. | | | | 1.00 | | | | | ref. |
| Overweight | 1.15 | | | 0.77 - 1.72 | | | | 1.13 | | 0.76 - 1.68 | | | | 1.14 | | | | | 0.76 - 1.70 |
| Obese | 1.46 | | | 0.87 - 2.43 | | | | 1.40 | | 0.84 - 2.33 | | | | 1.40 | | | | | 0.84 - 2.33 |
| *Number of subjects N* | 5797 | | |  | | | | 5793 | |  | | | | 5784 | | | | |  |
| **Low personal income (*time)** | | | | | | |  | | | | |  | | | | | |  | |
| Normal weight | 1.00 | | | ref. | | | |  | |  | | | |  | | | | |  |
| Overweight | 1.17 | | | 0.98 - 1.39 | | | |  | |  | | | |  | | | | |  |
| Obese | 1.34* | | | 1.06 - 1.69 | | | |  | |  | | | |  | | | | |  |
| *Number of subjects N* | *4658* | | |  | | | |  | |  | | | |  | | | | |  |
| Socioeconomic disadvantage measures as outcome variables  CI, confidence interval.  Weight category defined by body mass index (normal weight BMI 18.50-24.99, overweight BMI 25.00-29.99 and obese BMI >30.00) | | | | | | | | | | | | | | | | | | |  |
| a Adjusted for age. | | |  | | |  | | | | |  | | | |  | | | |  |
| b Additionally adjusted for marital status as time variant | | | | | | | | | | | | | | | | | | |  |
| c Additionally adjusted for employment status as time variant | | | | | | | | | | | | | | | | | | |  |
