# Supplementary material for: Obesity and socioeconomic disadvantage in midlife female public sector employees: a cohort study
Source: BMC Public Health. 2017 Oct 24;17:842. doi: 10.1186/s12889-017-4865-8 (PMC5655943; doi:10.1186/s12889-017-4865-8)
Supplement: Supplementary file 3 — Odds ratios (95% confidence intervals) for associations time variant body weight status and socioeconomic disadvantage at Phases 1–3 among women, repeated measures analysis (GEE), the Helsinki Health Study, Finland, 2000–2012. (DOCX 77 kb) [file 12889_2017_4865_MOESM3_ESM.docx]

| **Supplement table 3. Odds ratios (95% confidence intervals) for associations time variant body weight status and socioeconomic disadvantage at Phases 1-3 among women, repeated measures analysis (GEE), the Helsinki Health Study, Finland, 2000-2012** | | | | | | | | | | | |
| --- | --- | --- | --- | --- | --- | --- | --- | --- | --- | --- | --- |
|  | **Model 1^a^** | | | | | **Model 2^b^** | | | | | |
| **Low household net income** | | Odds ratio | | | 95% CI | | | Odds ratio | | | 95% CI |
| Normal weight | | 1.00 | | | ref. | | | 1.00 | | | ref. |
| Overweight | | 1.01 | | | 0.93 – 1.10 | | | 1.12 | | | 1.00 – 1.24 |
| Obese | | 1.11 | | | 0.99 – 1.24 | | | 1.19 | | | 1.04 – 1.36 |
| *Model N* | | *6829* | | |  | | | *6827* | | |  |
| *QICC** | | *18158.05* | | |  | | | *11708.1* | | |  |
| **Income below poverty** | | |  | | | |  | | |  | |
| Normal weight | | 1.00 | | | ref. | | | 1.00 | | | ref. |
| Overweight | | 0.99 | | | 0.88 – 1.11 | | | 1.02 | | | 0.90 – 1.15 |
| Obese | | 1.15 | | | 1.00 – 1.33 | | | 1.15 | | | 0.99 – 1.33 |
| *Model N* | | *6829* | | |  | | | *6827* | | |  |
| *QICC* | | *11957.11* | | |  | | | *10656.42* | | |  |
| **Frequent economic difficulties** | | | |  | | | | |  | | |
| Normal weight | | 1.00 | | | ref. | | | 1.00 | | | ref. |
| Overweight | | 1.17 | | | 1.06 – 1.28 | | | 1.19 | | | 1.08 – 1.31 |
| Obese | | 1.52 | | | 1.35 – 1.71 | | | 1.53 | | | 1.36 – 1.73 |
| *Model N* | | *6857* | | |  | | | *6853* | | |  |
| *QICC* | | *16940.26* | | |  | | | *16378.04* | | |  |
| **Low household wealth** | | |  | | | |  | | |  | |
| Normal weight | | 1.00 | | | ref. | | | 1.00 | | | ref. |
| Overweight | | 1.29 | | | 1.14 – 1.45 | | | 1.30 | | | 1.15 – 1.46 |
| Obese | | 1.68 | | | 1.45 – 1.94 | | | 1.68 | | | 1.45 – 1.95 |
| *Model N* | | *5718* | | |  | | | *5714* | | |  |
| *QICC* | | *9140.57* | | |  | | | *9029.38* | | |  |
| **Low personal income** | | |  | | | |  | | |  | |
| Normal weight | | 1.00 | | | ref. | | |  | | |  |
| Overweight | | 1.09 | | | 0.97 – 1.23 | | |  | | |  |
| Obese | | 1.25 | | | 1.07 – 1.45 | | |  | | |  |
| *Model N* | | *4626* | | |  | | |  | | |  |
| *QICC* | | *9260.90* | | |  | | |  | | |  |
| CI. Confidence interval.  Weight category defined by body mass index (normal weight BMI 18.50-24.99, overweight BMI 25.00-29.99 and obese BMI >30.00) | | | | | | | | | | | |
| *Corrected Quasi Likelihood under Independence Model Criterion (in smaller-is-better form) | | | | | | | | | | | |
| a Adjusted for age and education level | | | | | | | | | | | |
| b Additionally adjusted for marital status as a time variant covariate | | | | | | | | | | | |
