# Supplementary material for: Obesity and socioeconomic disadvantage in midlife female public sector employees: a cohort study
Source: BMC Public Health. 2017 Oct 24;17:842. doi: 10.1186/s12889-017-4865-8 (PMC5655943; doi:10.1186/s12889-017-4865-8)
Supplement: Supplementary file 4 — Response rates at Phases 2 and 3 by baseline body weight status and indicators of socioeconomic disadvantage among women, the Helsinki Health Study, Finland, 2000–2012. (DOCX 65 kb) [file 12889_2017_4865_MOESM4_ESM.docx]

| **Supplement table 4. Response rates at Phases 2 and 3 by baseline body weight status and indicators of socioeconomic disadvantage among women, the Helsinki Health Study, Finland, 2000-2012** | | | | | | | | | | | | | |
| --- | --- | --- | --- | --- | --- | --- | --- | --- | --- | --- | --- | --- | --- |
| **Baseline characteristic** | | **Phase 2** | | | | | **p value** | | **Phase 3** | | | | **p value** |
| **Weight status** | | % | | n |  | | | % | | n | |  | |
| Normal weight | | 84.5 | | 3140 |  | | | 79.6 | | 2956 | |  | |
| Overweight | | 83.9 | | 1847 |  | | | 79.2 | | 1745 | |  | |
| Obese | | 82.5 | | 823 | 0.300 | | | 74.1 | | 739 | | 0.001 | |
| **Marital status** |  | |  | | |  | | | | |  | | |
| Living alone | | 82.5 | | 1854 |  | | | 76.9 | | 1728 | |  | |
| Married or cohabiting | | 84.8 | | 3946 | 0.016 | | | 79.5 | | 3700 | | 0.014 | |
| **Low household income** | |  | |  |  | | |  | |  | |  | |
| No | | 84.7 | | 4207 |  | | | 79.8 | | 3963 | |  | |
| Yes | | 83.0 | | 1455 | 0.088 | | | 76.3 | | 1339 | | 0.002 | |
| **Poverty** | |  | |  |  | | |  | |  | |  | |
| No | | 84.3 | | 4970 |  | | | 79.1 | | 4666 | |  | |
| Yes | | 83.9 | | 686 | 0.759 | | | 77.0 | | 630 | | 0.166 | |
| **Frequent economic difficulties** | |  | |  |  | | |  | |  | |  | |
| No | | 85.0 | | 4513 |  | | | 80.3 | | 4263 | |  | |
| Yes | | 81.3 | | 1266 | <0.001 | | | 73.2 | | 1140 | | <0.001 | |
| **Low personal income** | |  | |  |  | | |  | |  | |  | |
| No | | 87.8 | | 3234 |  | | | 82.2 | | 3028 | |  | |
| Yes | | 83.7 | | 757 | 0.001 | | | 79.5 | | 719 | | 0.062 | |
| P values: differences between baseline groups (from χ2 test)  Weight category defined by body mass index (normal weight BMI 18.50-24.99, overweight BMI 25.00-29.99 and obese BMI >30.00) | | | | | | | | | | | | | |
